# Numerical Study on Thermal Stress of High Temperature Proton Exchange Membrane Fuel Cells during Start-Up Process

**DOI:** 10.3390/membranes13020215

**Published:** 2023-02-09

**Authors:** Shian Li, Chengdong Peng, Qiuwan Shen, Yuanzhe Cheng, Chongyang Wang, Guogang Yang

**Affiliations:** Marine Engineering College, Dalian Maritime University, Dalian 116026, China

**Keywords:** HT-PEMFCs, PBI membrane, start-up, three-dimensional mathematical model, flow arrangement, thermal stress

## Abstract

High-temperature proton-exchange membrane fuel cells (HT-PEMFCs) with phosphoric-doped polybenzimidazole (PBI) membranes have a higher operating temperature compared to the PEMFCs operating below 373.15 K. The fuel cell is first heated from room temperature to the minimum operating temperature to avoid the generation of liquid water. The existence of liquid water can result in the loss of phosphoric acid and then affect the cell performance. In this study, the start-up process of HT-PEMFCs is numerically studied by establishing a three-dimensional non-isothermal mathematical model. Preheated gas is supplied into gas flow channels to heat the fuel cell, and then voltage load is applied to accelerate the start-up process. Effects of voltage (0.9 V, 0.7 V and 0.5 V) and flow arrangement (co-flow and counter flow) on temperature, current density, proton conductivity and stress distributions of fuel cells are examined. It is found that the maximum stress is increased when a lower voltage is adopted, and the counter-flow arrangement provides a more uniform stress distribution than that of co-flow arrangement.

## 1. Introduction

In response to the increasing pressure on carbon emissions, the choice of proton exchange membrane fuel cells (PEMFCs) as energy conversion devices is considered a practical approach [1]. Meanwhile, renewable energies such as solar/wind energy are also very popular in the global decarbonization context. However, the additional employment of energy storage/generators is needed to improve the utilization rate and stability of renewable energies. This is because they are unstable and intermittent during the generation process [2]. PEMFCs are known for high power density, fast response speed, cleanliness, and high conversion efficiency. Low-temperature PEMFCs (LT-PEMFCs) require complex water and thermal management strategies to ensure their performance. In order to simplify the needed auxiliary equipment, high-temperature PEMFCs (HT-PEMFCs) are proposed, which have many advantages, including faster electrochemical kinetics, easy water management, and higher tolerance to impurities [3,4,5,6]. HT-PEMFCs with phosphoric-doped polybenzimidazole (PBI) membranes have a higher operating temperature than LT-PEMFCs.

There are many numerical studies related to HT-PEMFCs in the available literature. Li et al. [7] studied effect of membrane phosphoric acid doping level on transport characteristics and cell performance. It was reported that cell performance is increased with the increase in doping level. Acid doping can improve the conductivity of PBI membrane, but it will not increase indefinitely with the increase in doping level. The amount of phosphoric acid was optimized to achieve cell performance improvement by performing experimental studies, and then the optimum amount was obtained [8]. Fuel utilization and performance of HT-PEMFCs were estimated when different stoichiometric ratios were adopted [9]. Effect of temperature on transport phenomena and performance of HT-PEMFCs were studied [10,11]. In addition, HT-PEMFCs with different flow field designs [12,13,14] and membranes [15,16] and were extensively investigated.

The generated liquid water in HT-PEMFCs can result in the loss of phosphoric acid, and then the cell performance is degraded [17]. Therefore, fuel cell is firstly heated from room temperature to the minimum operating temperature of 393.15 K [18]. In order to fulfill the abovementioned start-up process, different heating methods have already been proposed. Commonly used start-up strategies of HT-PEMFCs include direct electrical heating, reactant heating, coolant heating, exothermic reaction heating, and ohmic heating [19,20,21]. The direct electric heating method relies on external electric heaters to heat the endplates. For the reactant heating, the temperature of the fuel cell is increased by supplying preheated reactants to the gas flow channels. For coolant heating, the preheated coolant in the cooling channels is used to heat the fuel cell. The total reaction of the fuel cell is an exothermic process. Ohmic heating relies on the heat generated by the cell operating at a high current density. There are many studies on fuel cell start-up strategies. Zhang et al. [18] simulated the influence of different flow velocities, flow configurations, and load currents on start-up time and temperature distribution. It was concluded that load current strategy can effectively reduce start-up time. Rasheed et al. [22] studied the effect of inlet gas heating and applied voltage on the fuel cell start-up process and found that the applied voltage significantly shortened the start-up time. Choi et al. [23] combined three heating methods of coolant heating, reaction heating and inlet gas heating to study the start-up process. The results showed that the combination of coolant and reaction heating provided a better performance. Wang et al. [24] confirmed that the combination of coolant and reaction heating is an effective heating strategy after comparing various start-up strategies.

The membrane is a key component of PEMFCs. The Nafion membrane is commonly used in LT-PEMFCs, and it has good mechanical properties [25]. The backbone of PBI is not strong enough, so stress will cause a more significant degradation problem in PBI membranes. Perfluorosulfonic acid membranes or sulfonated hydrocarbon membranes based on a rigid skeleton are difficult to use in HT-PEMFCs; this is because the sulfonic group will degrade at a high temperature of 403.15 K [26]. Compared to other components of fuel cells, the membrane has an enormous thermal expansion coefficient. Fragile membrane structures are more susceptible to damage under the influence of thermal stress. Therefore, it is necessary to perform numerical analyses of the thermal stress during the start-up process. Oh et al. [27] studied thermal stress during the clamping process, start-up process, and operation of HT-PEMFCs using the three-dimensional finite element method. The results showed that during the start-up process, a higher level of thermal stress occurs inside the cell. Varghese et al. [28] studied the thermal gradient and thermal stress of HT-PEMFCs during the start-up process with several heating methods. It was concluded that improper start-up strategies can affect cell performance.

Thermal stress distribution on the membrane of HT-PEMFC during the start-up process can significantly affect the operation safety and long-term stability. However, studies on thermal stress of HT-PEMFC are still very few. So, it is necessary to perform analyses on thermal stress of fuel cells during the start-up process. In this study, the start-up process of a fuel cell is numerically studied by establishing a three-dimensional non-isothermal mathematical model. Prior to numerical simulations, model validation and a mesh independence test were carried out. Hydrogen and air were preheated and then provided in the anode and cathode gas flow channels, respectively. The average membrane temperature gradually increased from 293.15 K to 393.15 K, and then voltage was applied to speed up the heating process. The stress distribution of membranes under different voltages (0.9 V, 0.7 V and 0.5 V) and flow arrangements (co-flow and counter flow) is presented and compared. This study can improve the understanding of thermal stress of HT-PEMFCs during start-up process. In addition, the corresponding temperature, current density and proton conductivity distributions are analyzed.

## 2. Model Description

**Figure 1:**
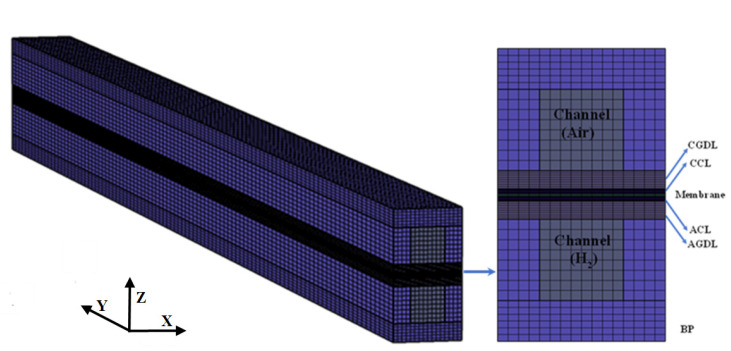
The computational domain and mesh used in this study.

In this study, a single straight channel is selected as the computational domain, which is composed of bipolar plates (BPs), gas channels (GCs), gas diffusion layers (GDLs), catalyst layers (CLs), and membrane (MEM). Table 1 lists relevant geometric parameters and material properties of the fuel cell. The computational domain and the corresponding mesh configuration are shown in Figure 1.

### 2.1. Governing Equations

The assumptions used in this mathematical model are as follows: the flow is laminar; ideal gas law is adopted; and the porous structure is isotropic.

The numerical model consists of mass, momentum, species, energy, charge and solid mechanics conservation equations.

Mass equation:

(1) $$\frac{\partial \varepsilon \rho }{\partial t}+\nabla \left(\vec{\rho u}\right)=S_{m}$$

where ε is the porosity of the porous medium, and ρ represents the mixture gas density calculated by the law of ideal gas. S_m_ is the mass source term. In addition, the source or sink terms used in this paper are shown in Table 2. 

Momentum conservation:

(2) $$\frac{\rho }{\varepsilon }\left(\frac{\partial \vec{u}}{\partial t}+\left(\nabla \cdot \vec{u}\right)\frac{\vec{u}}{\varepsilon }\right)=\nabla \cdot \left[\frac{\mu }{\varepsilon }\left(\nabla \vec{u}+{\left(\nabla \vec{u}\right)}^{T}\right)-\frac{2\mu }{3\varepsilon }\left(\nabla \cdot \vec{u}\right)I\right]-\nabla P+S_{u}$$

where I is the identity matrix, μ is the dynamic viscosity of gas mixture, and K is the permeability. The physical parameters of the relevant gases are shown in Table 3.

Specie conservation:

(3) $$\frac{\partial }{\partial t}\left({\varepsilon \rho Y}_{i}\right)+\nabla \cdot \left({\rho \vec{u}Y}_{i}\right)=\nabla \cdot \left({\rho D}_{i}^{\mathrm{eff}}\nabla Y_{i})\right.+S_{i}$$

where D_i_ is the diffusion coefficient of gas components. Considering the porosity and curvature of porous media, the Bruggeman correlation can be used to modify mass diffusivities [29]:

(4) $$D_{i}^{\mathrm{eff}}=\varepsilon^{1.5}D_{i}\;$$

Charge conservation:

(5) $$\nabla \cdot \left(\sigma_{\mathrm{eff},\mathrm{ele}}\nabla \phi_{\mathrm{ele}}\right){+S}_{\mathrm{ele}}=0\;$$

(6) $$\nabla \cdot \left(\sigma_{\mathrm{eff},p}\nabla \phi_{p}\right){+S}_{p}=0$$

where σ_eff,ele_ and σ_eff,p_ are the effective electron conductivity and the effective proton conductivity corrected by the Bruggeman correlation [30].

(7) $$\;\sigma_{\mathrm{eff},\mathrm{ele},\mathrm{CL}}={\left(1-\varepsilon -\omega \right)}^{1.5}\sigma_{\mathrm{ele},\mathrm{CL}}$$

(8) $${\;\sigma }_{\mathrm{eff},p,\mathrm{CL}}=\omega^{1.5}\sigma_{p,\mathrm{CL}}$$

(9) $$\sigma_{\mathrm{eff},\mathrm{ele},\mathrm{GDL}}={\left(1-\varepsilon \right)}^{1.5}\sigma_{\mathrm{ele},\mathrm{GDL}}$$

The conductivity of the electrolyte is a function of temperature. The specific formula is as follows [31]:

(10) $$\sigma_{p}=\frac{100}{T}\exp\left[8.02-\left(\frac{2605.6-70.1\mathrm{DL}}{T}\right)\right]$$

where DL is the doping level of membrane phosphoric acid. In this study, the value of DL (DL = 11) is taken from the reference value given by Devrim et al. [32] in the experiment.

On the other hand, the source term in the charge conservation equation is related to the transfer current density j_a/c_, which reflects the local reaction rate. Additionally, the value of current density can be determined by Equations (11)–(14) [33,34]:

(11) $$j_{a}={\mathrm{ai}}_{0,a}{\left(\frac{C_{H_{2}}}{C_{H_{2},\mathrm{ref}}}\right)}^{\frac{1}{2}}\left(e^{\frac{{\alpha F\eta }_{a}}{\mathrm{RT}}}-e^{\frac{-{\alpha F\eta }_{a}}{\mathrm{RT}}}\right)$$

(12) $$j_{c}=-{\mathrm{ai}}_{0,c}\left(\frac{C_{O}{}_{2}}{C_{O_{2},\mathrm{ref}}}\right)\cdot \left(e^{\frac{{\alpha F\eta }_{c}}{\mathrm{RT}}}-e^{\frac{-{\alpha F\eta }_{c}}{\mathrm{RT}}}\right)$$

(13) $$i_{0,a}=i_{0,a}^{\mathrm{ref}}\cdot \exp\left[-1400\left(\frac{1}{T}-\frac{1}{353.15}\right)\right]$$

(14) $$i_{0,c}=i_{0,c}^{\mathrm{ref}}\cdot \exp\left[-7900\left(\frac{1}{T}-\frac{1}{353.15}\right)\right]$$

In the above formula, a is the ratio of reaction surface to CL volume, α is the transfer coefficient. The relevant parameters for the electrochemical equations are listed in Table 4. In addition, the overpotential in the electrochemical reaction, η, as expressed below:

(15) $$\eta_{a}=\phi_{\mathrm{ele}}-\phi_{\mathrm{ion}}-E_{\mathrm{eq},a}\;\left(\mathrm{in}\;\mathrm{ACL}\right)\;$$

(16) $$\eta_{c}=\phi_{\mathrm{ele}}-\phi_{\mathrm{ion}}-E_{\mathrm{eq},c}\;\left(\mathrm{in}\;\mathrm{CCL}\right)$$

where the thermodynamic equilibrium potential of cathode and anode can be expressed by the following equation [35]:

(17) $$E_{\mathrm{eq},a}=0\;\left(\mathrm{in}\;\mathrm{ACL}\right)$$

(18) $$E_{\mathrm{eq},c}=1.1669-0.241\times 10^{-3}\left(T-373.15\right)\;\left(\mathrm{in}\;\mathrm{CCL}\right)$$

Energy conservation:

(19) $$\frac{\partial T}{\partial t}\left({\left({\rho C}_{p}\right)}^{\mathrm{eff}}T\right)+\nabla \cdot \left({\left({\rho C}_{p}\right)}^{\mathrm{eff}}\vec{u}T\right)=\nabla \left(k^{\mathrm{eff}}\nabla T\right)+S_{T}\;$$

where C_p_ is heat capacity, and k is thermal conductivity. Considering the influence of porosity (ε) and electrolyte volume (ω) in porous areas, the values of C_p_ and k need to be corrected. The specific heat capacity and thermal conductivity of the gas mixture are given by [36]:

(20) $$k_{g}=\sum_{i}\frac{Y_{i}k_{i}}{\sum_{j=1}Y_{j}\sqrt{\frac{M_{j}}{M_{i}}}}\;$$

(21) $${\left(C_{P}\right)}_{g}=\underset{i}{\sum }Y_{i}{\left(C_{P}\right)}_{i}$$

where Y_i_ and M_i_ are mass fractions and molar weights of gas components.

Solid mechanics models and equations:

It is assumed that fuel cell components are linear elastic and isotropic materials. The stress relationship satisfies the following formula [37]:

(22) $$\sigma_{\mathrm{tol}}=\sigma_{0}+\sigma_{\mathrm{ext}}+{D\varepsilon }_{\mathrm{el}}$$

where σ_tol_ is the total stress, σ_0_ and σ_ext_ are the prestress and the external stress, respectively, and their values are 0. Hence, the total strain at any position is contributed by thermal strain ε_th_ and elastic strain ε_el_.

(23) $$\varepsilon =\varepsilon_{\mathrm{th}}+\varepsilon_{\mathrm{el}}+\varepsilon_{0}+\varepsilon_{\mathrm{ext}}=\varepsilon_{\mathrm{th}}+\varepsilon_{\mathrm{el}}$$

(24) $$\varepsilon_{\mathrm{th}}=\alpha \left(T-T_{\mathrm{ref}}\right)$$

where α presents the thermal expansion coefficient of the material. D is the stiffness modulus, and the equation is shown below:

(25) $$\;D=\frac{E}{\left(1+v\right)\left(1-2v\right)}\;\begin{array}{c} \times \left[\begin{array}{cccccc} 1-U & v & v & 0 & 0 & 0\\ v & 1-v & v & 0 & 0 & 0\\ v & v & i-v & 0 & 0 & 0\\ 0 & 0 & 0 & (1-2v)/2 & 0 & 0\\ 0 & 0 & 0 & 0 & \left(1-2v\right)/2 & 0\\ 0 & o & 0 & 0 & 0 & \left(1-2v\right)/2 \end{array}\right]\;\;\; \end{array}$$

where E is Young’s modulus, v is Poisson’s ratio, and the stress–strain relationship can be corrected to the following formula:

(26) $$\left[\begin{array}{c} \sigma_{\mathrm{xx}}\\ \sigma_{\mathrm{yy}}\\ \sigma_{\mathrm{zz}}\\ \sigma_{\mathrm{yz}}\\ \sigma_{\mathrm{xz}}\\ \sigma_{\mathrm{xy}} \end{array}\right]=D\left[\begin{array}{c} \varepsilon_{\mathrm{xx}}\\ \varepsilon_{\mathrm{yy}}\\ \varepsilon_{\mathrm{zz}}\\ \varepsilon_{\mathrm{yz}}\\ \varepsilon_{\mathrm{xz}}\\ \varepsilon_{\mathrm{xy}} \end{array}\right]-\beta \Delta T\left[\begin{array}{c} 1\\ 1\\ 1\\ 0\\ 0\\ 0 \end{array}\right]$$

(27) $$\beta =\frac{\alpha E}{1-2v}$$

Von Mises yield criterion is used to modify the stress, and the equivalent stress of the material could be expressed by:

(28) $$\sigma_{v}=\frac{1}{\sqrt{2}}\sqrt{{\left(\sigma_{\mathrm{xx}}-\sigma_{\mathrm{yy}}\right)}^{2}+{\left(\sigma_{\mathrm{yy}}-\sigma_{\mathrm{zz}}\right)}^{2}+{\left(\sigma_{\mathrm{zz}}-\sigma_{\mathrm{xx}}\right)}^{2}+3\left(\sigma_{\mathrm{xx}}{}^{2}+\sigma_{\mathrm{xx}}{}^{2}+\sigma_{\mathrm{zx}}{}^{2}\right)}\;$$

### 2.2. Numerical Procedure and Boundary Condition

The HT-PEMFC mathematical model is established using finite element-based commercial software COMSOL Multiphysics. Inflow velocity and the corresponding mass fraction were specified at the inlet of GCs. Outflow conditions were used at the outlet of GCs with a constant pressure of 1.0 atm. and zero flux boundary conditions for all other external walls. Hydrogen and air at a temperature of 433.15 K were supplied into the anode and cathode GCs to heat the fuel cell. When the voltage was applied, the anode current collector was set to 0, and the cathode current collector was equal to the fuel cell operating voltage V_cell_. The corresponding anode and cathode velocities can be calculated by the following equations [38]:

(29) $$u_{\mathrm{in},a}=\xi_{a}I_{\mathrm{ave}}Y_{H_{2}}\mathrm{RT}/\left(2{\mathrm{FPA}}_{\mathrm{in}}\right)\;$$

(30) $$u_{\mathrm{in},c}=\xi_{c}I_{\mathrm{ave}}Y_{O_{2}}\mathrm{RT}/\left(4{\mathrm{FPA}}_{\mathrm{in}}\right)$$

where u_in,a/c_ is the velocity, ξ_a/c_ is the stoichiometric ratio, I_ave_ is the reference average current density, and A_in_ is the cross-sectional area of GCs. 

**Figure 2:**
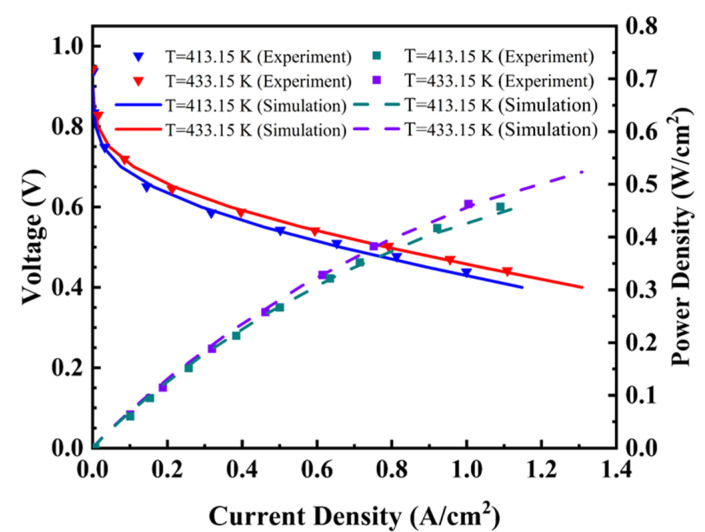
The predicted results compared with the experimental data.

**Figure 3:**
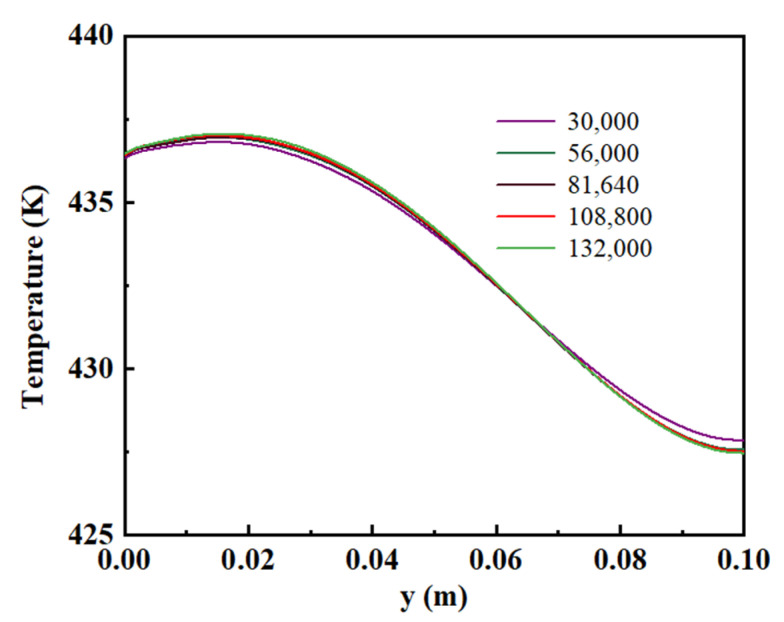
The mesh independence test.

The model validation is performed. The polarization and power density curves reported by Devrim et al. [32] are compared with the results produced by the numerical simulations to verify the accuracy of the mathematical model. The calibration results are shown in Figure 2. Under two working conditions, the model curves fit well with the experimental data, and these prove the usability of the model. The mesh independent test is carried out by comparing the temperature distribution in the membrane when different grid numbers are used in the numerical simulations. As shown in Figure 3, the grid numbers of 30,000, 56,000, 81,640, 108,800 and 132,000 are used to perform the numerical simulations, and then the number 108,800 is selected for the following calculations.

## 3. Results and Discussion

**Figure 4:**
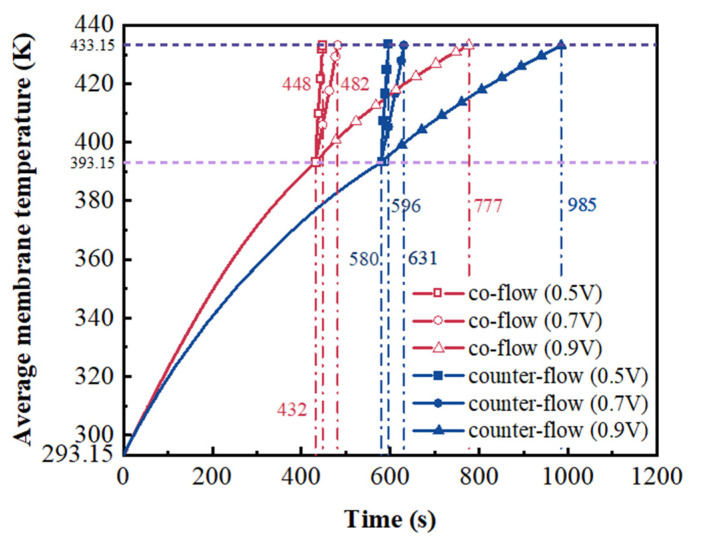
The variation of average membrane temperature of fuel cells at different voltages.

The fuel cell was heated form 293.15 K to the operating temperature of 433.15 K. Hydrogen and air were preheated to 433.15 K and then provided into the anode and cathode GCs with co-flow and counter-flow arrangements, respectively. Consequently, the fuel cell can be heated by the supplied gas. The average membrane temperature is firstly heated from 293.15 K to 393.15 K and then heated from 393.15 K to 433.15 K. When the average membrane temperature reaches 393.15 K, voltage is applied to speed up the start-up process. The variation of average membrane temperature at different voltages of 0.9 V, 0.7 V and 0.5 V is presented in Figure 4. The time required to reach the average membrane temperature of 393.15 K at co-flow and counter-flow arrangements is 432 s and 580 s, respectively. The co-flow arrangement can reduce the required time in comparison with the counter-flow arrangement. For the co-flow arrangement, the time consumed in the second stage at the voltages of 0.9 V, 0.7 V and 0.5 V is 345 s, 50 s, and 16 s, respectively. For the counter-flow arrangement, the time consumed in the second stage at the voltages of 0.9 V, 0.7 V and 0.5 V is 405 s, 51 s, and 16 s, respectively. It is found that the required time can be decreased by decreasing the voltage applied. The generation heat caused by the electrochemical reactions is increased with decreased in the voltage. This can be explained by the polarization curve. When the voltage decreases, the current density increases. The source term of energy equation is increased with increasing current density [18]. This means that more heat is generated when a lower voltage is applied.

**Figure 5:**
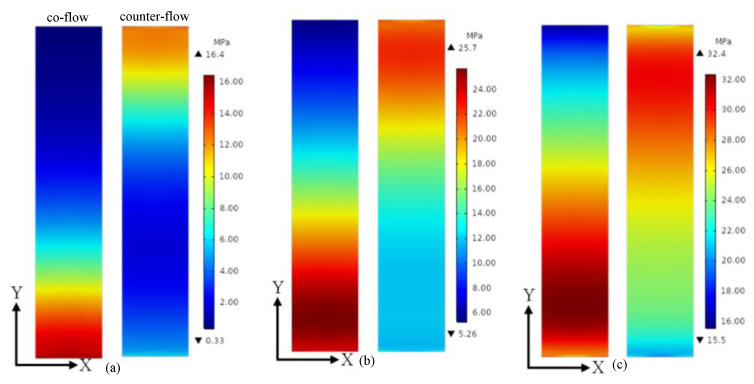
The stress distributions on the middle plane of the membrane at different average membrane temperature: (**a**) 313.15 K; (**b**) 353.15 K; (**c**) 393.15 K.

The maximum stress in the fuel cell is concentrated on the membrane. This is because that the membrane has the maximum thermal expansion coefficient among the cell components [28]. Therefore, the stress distribution in the membrane is presented and compared in the following section. The stress distributions on the middle plane of the membrane at the average membrane temperature of 313.15 K, 353.15 K and 393.15 K are presented in Figure 5. The stress distribution is significantly affected by the flow arrangement. When the average membrane temperature is 313.15 K, the minimum stresses of co-flow and counter-flow arrangements are 0.33 Mpa and 1.99 Mpa, and the maximum stresses of them are 16.45 Mpa and 12.79 Mpa, respectively. When the average membrane temperature is 353.15 K, the minimum stresses of co-flow and counter-flow arrangements are 5.26 Mpa and 11.72 Mpa, and the maximum stresses of them are 25.67 Mpa and 22.36 Mpa, respectively. When the average membrane temperature is 393.15 K, the minimum stresses of co-flow and counter-flow arrangements are 15.53 Mpa and 19.91 Mpa, and the maximum stresses of them are 32.35 Mpa and 30.17 Mpa, respectively. The maximum stress is increased with the increasing temperature. It is also found that a higher maximum stress is provided by the co-flow arrangement and a smaller variation of stress is obtained by the counter-flow arrangement. The counter-flow arrangement is better for the operation safety and long-term stability of fuel cells. However, more time is needed for the start-up process, as discussed in Figure 4.

**Figure 6:**
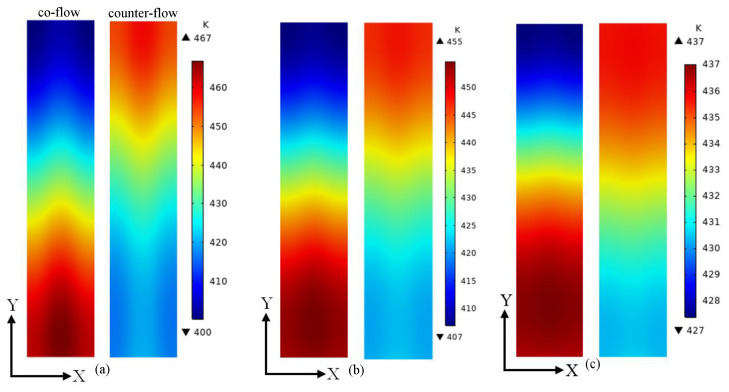
The temperature distributions on the middle plane of the membrane at different voltages: (**a**) 0.5 V; (**b**) 0.7 V; (**c**) 0.9 V.

When the average membrane temperature reaches 393.15 K, voltage is applied to speed up the start-up process. The temperature, current density, proton conductivity and stress distributions of the membrane are given and analyzed when the average membrane temperature is 433.15 K. Figure 6 gives the temperature distributions on the middle plane of the membrane at the voltages of 0.5 V, 0.7 V and 0.9 V. It is observed that the temperature distribution is influenced by the flow arrangement. The maximum temperature is decreased and the minimum temperature is increased when the voltage is increased from 0.5 V to 0.9 V. This is because more heat is generated when a lower voltage is applied. When the voltage is 0.5 V, the minimum temperature of co-flow and counter-flow arrangements is 400.30 K and 416.61 K, and the maximum temperature of them is 466.98 K and 458.24 K, respectively. When the voltage is 0.7 V, the minimum temperature of co-flow and counter-flow arrangements is 406.96 K and 421.13 K, and the maximum temperature of them is 454.76 K and 447.98 K, respectively. When the voltage is 0.9 V, the minimum temperature of co-flow and counter-flow arrangements is 427.39 K and 430.44 K, and the maximum temperature of them is 437.01 K and 435.78 K, respectively. The temperature difference is increased when the voltage is decreased from 0.9 V to 0.5 V. This indicates that a more uniform temperature distribution can be obtained when a higher voltage is used. Meanwhile, a smaller temperature difference is provided by the counter-flow arrangement when the same voltage is adopted. A smaller temperature difference means a more uniform temperature distribution, which is good for the membrane.

**Figure 7:**
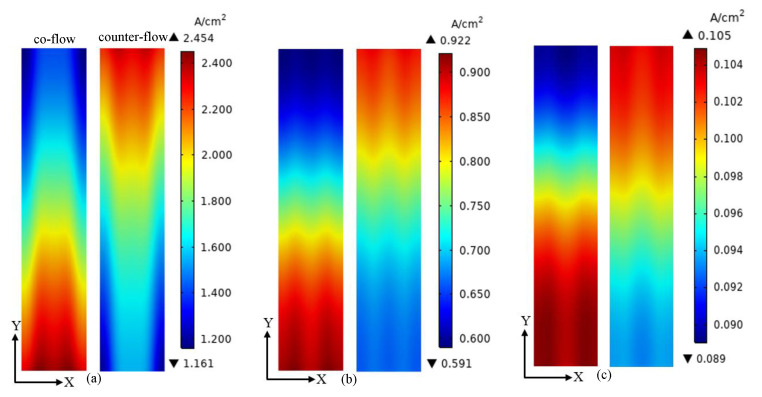
The current density distributions on the middle plane of the membrane at different voltages: (**a**) 0.5 V; (**b**) 0.7 V; (**c**) 0.9 V.

The current density distributions on the middle plane of the membrane at the voltages of 0.5 V, 0.7 V and 0.9 V are presented in Figure 7. The current density distribution is greatly affected by the voltage and flow arrangement. When the voltage is 0.5 V, the minimum current density of co-flow and counter-flow arrangements is 1.161 A/cm^2^ and 1.208 A/cm^2^, and the maximum current density of them is 2.454 A/cm^2^ and 2.363 A/cm^2^, respectively. When the voltage is 0.7 V, the minimum current density of co-flow and counter-flow arrangements is 0.591 A/cm^2^ and 0.662 A/cm^2^, and the maximum current density of them is 0.922 A/cm^2^ and 0.886 A/cm^2^, respectively. When the voltage is 0.9 V, the minimum current density of co-flow and counter-flow arrangements is 0.089 A/cm^2^ and 0.093 A/cm^2^, and the maximum current density of them is 0.105 A/cm^2^ and 0.104 A/cm^2^, respectively. According to the polarization curve, the current density is increased with the decreasing voltage. Meanwhile, the uniformity of current density is increased with increasing voltage. A more uniform current density distribution is good for the membrane.

**Figure 8:**
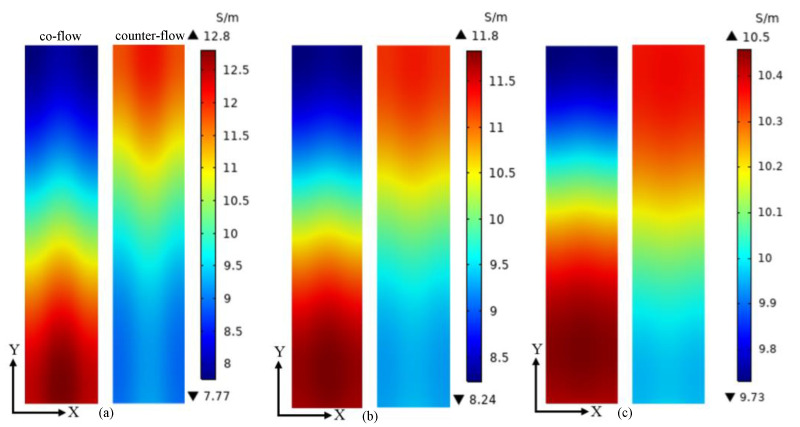
The proton conductivity distributions on the middle plane of membrane at different voltages: (**a**) 0.5 V; (**b**) 0.7 V; (**c**) 0.9 V.

The proton conductivity on the middle plane of membrane at the voltages of 0.5 V, 0.7 V and 0.9 V are given in Figure 8. The proton conductivity has the same distribution as the temperature, because it is temperature-dependent. When the voltage is 0.5 V, the minimum proton conductivity of co-flow and counter-flow arrangements is 7.77 S/m and 8.93 S/m, and the maximum proton conductivity of them is 12.81 S/m and 12.11 S/m, respectively. When the voltage is 0.7 V, the minimum proton conductivity of co-flow and counter-flow arrangements is 8.24 S/m and 9.26 S/m, and the maximum proton conductivity of them is 11.84 S/m and 11.31 S/m, respectively. When the voltage is 0.9 V, the minimum proton conductivity of co-flow and counter-flow arrangements is 9.73 S/m and 9.96 S/m, and the maximum proton conductivity of them is 10.46 S/m and 10.36 S/m, respectively. The uniformity of proton conductivity is increased for both flow arrangements when the voltage is increased from 0.5 V to 0.9 V. Under the same voltage, a more uniform proton conductivity distribution is obtained by the counter-flow arrangement. A more uniform proton conductivity distribution is good for the membrane.

**Figure 9:**
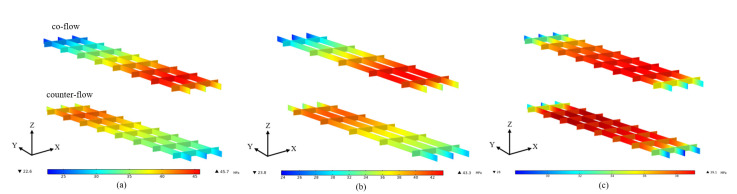
The stress distributions of membrane at different voltages: (**a**) 0.5 V; (**b**) 0.7 V; (**c**) 0.9 V.

Figure 9 displays the stress distribution of the membrane at the voltages of 0.5 V, 0.7 V and 0.9 V. It can be observed that the stress distribution is strongly affected by the voltage and flow arrangement. When the voltage is 0.5 V, the minimum stresses of co-flow and counter-flow arrangements are 22.63 Mpa and 26.19 Mpa, and the maximum stresses of them are 45.74 Mpa and 43.06 Mpa, respectively. When the voltage is 0.7 V, the minimum stresses of co-flow and counter-flow arrangements are 23.82 Mpa and 26.89 Mpa, and their maximum stresses are 43.33 Mpa and 41.17 Mpa, respectively. When the voltage is 0.9 V, the minimum stresses of co-flow and counter-flow arrangements are 28.01 Mpa and 28.67 Mpa, and their maximum stresses are 39.13 Mpa and 38.73 Mpa, respectively. This indicates that a more uniform stress distribution can be obtained when a higher voltage is used. Meanwhile, a smaller stress difference is provided by the counter-flow arrangement when the same voltage is adopted. A smaller stress difference means a more uniform stress distribution, which is good for the membrane. The counter-flow arrangement and a higher voltage are good for the operation safety and long-term stability of fuel cells. 

## 4. Conclusions

In this study, a three-dimensional non-isothermal mathematical model is established to study the start-up process of HT-PEMFCs. The stress distribution of membranes under different voltages (0.9 V, 0.7 V and 0.5 V) and flow arrangements (co-flow and counter flow) is presented and compared. The corresponding temperature, current density and proton conductivity distributions are also analyzed. According to the obtained results, the following conclusions are drawn.

The co-flow arrangement provides a faster start-up process in comparison with the counter-flow arrangement and the non-uniformity of stress in the membrane is also increased. The required time for the start-up process is decreased and the maximum stress is increased when a lower voltage is adopted. This is because more reactants are consumed, a higher current density is obtained, and more heat is produced when the voltage is decreased. Meanwhile, the non-uniformity of stress in the membrane is also increased. The stress magnitude and distribution is strongly affected by the start-up strategy and then the operation safety and long-term stability of fuel cells are also impacted. So, an appropriate start-up strategy should be carefully selected to avoid the effect of stress on cell performance and lifetime.

## Figures and Tables

**Table 1:** Parameters used in the mathematical model.

Parameter	Value	Units
Channel length/width/depth	$0{.1/\;1\times 10}^{-3}{/\;1\times 10}^{-3}$	m
Width of rib	${5\times 10}^{-5}$	m
Thickness of CL/GDL/M	${1\times 10}^{-5}/2{.25\times 10}^{-4}{/\;5\times 10}^{-5}$	m
Density	$\rho_{\mathrm{BP}/\mathrm{CL}/\mathrm{GDL}/\mathrm{MEM}}=2266/2145/1800/1300$	${\mathrm{kg}\;m}^{-3}$
Specific heat capacity	$C_{P_{\mathrm{BP}/\mathrm{CL}/\mathrm{GDL}/\mathrm{MEM}}}=1580/3300/710/1650$	${J\;\mathrm{kg}}^{-1}K^{-1}$
Thermal conductivity	$k_{\mathrm{BP}/\mathrm{CL}/\mathrm{GDL}/\mathrm{MEM}}=20/1.5/1.2/0.95$	${W\;m}^{-1}K^{-1}$
Electrical conductivity	$\sigma_{\mathrm{ele}}{}_{\mathrm{BP}/\mathrm{CL}/\mathrm{GDL}}=20,000/300/1250$	${S\;m}^{-1}$
Young’s modulus	$E_{\mathrm{BP}/\mathrm{CL}/\mathrm{GDL}/\mathrm{MEM}}=13,000/2953.2/6.3/5900$	MPa
Poisson’s ratio	$\nu_{\mathrm{BP}/\mathrm{CL}/\mathrm{GDL}/\mathrm{MEM}}=0.21/0.17/0.09/0.25$	
Thermal expansion coefficient	$\alpha_{\mathrm{BP}/\mathrm{CL}/\mathrm{GDL}/\mathrm{MEM}}=5/8.8/-0.8/37.7$	$10^{-6}K^{-1}$
Permeability	$K_{\mathrm{CL}/\mathrm{GDL}}=6.2{\times 10}^{-13}/6.2{\times 10}^{-12}$	$m^{2}$
Porosity	$\varepsilon_{\mathrm{CL}/\mathrm{GDL}}=0.3/0.6$	
Volume fraction of ionomer	0.21	

**Table 2:** Source terms of the governing equations.

Description	Units
$S_{m}{=S}_{H_{2}}{+S}_{O_{2}}{+S}_{H_{2}O}$ $S_{H_{2}}=\left\{\begin{array}{c} -\frac{j_{a}}{2F}M_{H_{2\;}}\left(\mathrm{in}\;\mathrm{ACL}\right)\\ \;0\;\left(\mathrm{in}\;\mathrm{other}\;\mathrm{zones}\right) \end{array}\right.{\;S}_{O_{2}}=\left\{\begin{array}{c} -\frac{j_{c}}{4F}M_{O_{2\;}\;}\left(\mathrm{in}\;\mathrm{CCL}\right)\\ \;0\;(\mathrm{in}\;\mathrm{other}\;\mathrm{zones}) \end{array}\right.$ $S_{H_{2}O}=\left\{\begin{array}{c} \frac{j_{c}}{2F}M_{H_{2}O_{}\;}\left(\mathrm{in}\;\mathrm{CCL}\right)\\ \;0\;(\mathrm{in}\;\mathrm{other}\;\mathrm{zones}) \end{array}\right.$	${\mathrm{kg}\;m}^{-3}{\;s}^{-1}$
$S_{u}=\left\{\begin{array}{c} -\left(\frac{\mu }{K}+\frac{S_{m}}{\varepsilon^{2}}\right)\vec{u}\;(\mathrm{in}\;\mathrm{GDL}\;\mathrm{and}\;\mathrm{CL})\\ \;0\;(\mathrm{in}\;\mathrm{other}\;\mathrm{zones}) \end{array}\right.$	${\mathrm{kg}\;m}^{-2}{\;s}^{-2}$
$S_{\mathrm{ele}}=\left\{\begin{array}{c} -j_{a}\;(\mathrm{in}\;\mathrm{ACL})\;\\ j_{c}\;(\mathrm{in}\;\mathrm{CCL})\\ 0\;(\mathrm{in}\;\mathrm{other}\;\mathrm{zones}) \end{array}\right.S_{\mathrm{ion}}=\left\{\begin{array}{c} \;\;\;j_{a}\;(\mathrm{in}\;\mathrm{ACL})\\ -j_{c}\;(\mathrm{in}\;\mathrm{CCL})\\ 0\;(\mathrm{in}\;\mathrm{other}\;\mathrm{zones}) \end{array}\right.$	${A\;m}^{-3}$
$S_{T}=\left\{\begin{array}{c} j_{a}\eta +\|{\nabla^{\emptyset }}_{\mathrm{ele}}\|{}^{2}\sigma_{\mathrm{ele}}^{\mathrm{eff}}+\|{\nabla^{\emptyset }}_{\mathrm{ion}}\|{}^{2}\sigma_{\mathrm{ion}}^{\mathrm{eff}}\;(\mathrm{in}\;\mathrm{ACL})\\ j_{c}\left(\eta +T\frac{{\mathrm{dE}}_{\mathrm{eq}}}{\mathrm{dT}}\right)+\|{\nabla^{\emptyset }}_{\mathrm{ele}}\|{}^{2}\sigma_{\mathrm{ele}}^{\mathrm{eff}}+\|{\nabla^{\emptyset }}_{\mathrm{ion}}\|{}^{2}\sigma_{\mathrm{ion}}^{\mathrm{eff}}\;(\mathrm{in}\;\mathrm{CCL})\\ \|{\nabla^{\emptyset }}_{\mathrm{ele}}\|{}^{2}\sigma_{\mathrm{ele}}^{\mathrm{eff}}\;(\mathrm{in}\;\mathrm{GDL}\;\mathrm{and}\;\mathrm{BP})\\ \|{\nabla^{\emptyset }}_{\mathrm{ion}}\|{}^{2}\sigma_{\mathrm{ion}}^{\mathrm{eff}}\;\left(\mathrm{in}\;\mathrm{membrane}\right)\\ 0\;(\mathrm{in}\;\mathrm{other}\;\mathrm{zones}) \end{array}\right.$	${W\;m}^{-3}$

**Table 3:** Parameters of gases used in the models.

Gas Parameters	Correlation/Value	Units
Stoichiometry ratio	$\xi_{a/c}=1.5/2.5$	
Average current density	$I_{\mathrm{ave}}=1.5$	${A\;\mathrm{cm}}^{-2}$
Anode inlet mass fraction	$Y_{H_{2}}=1$	
Cathode inlet mass fraction	$Y_{O_{2}{/N}_{2}}=0.233/0767$	
Reference temperature	$T_{\mathrm{ref}}=293.15$	K
Diffusion coefficient of species	$D_{H_{2}}=1.055{\times 10}^{-4}{\left(T/333.15\right)}^{1.5}\times (101325/P)$	$m^{2}{\;s}^{-1}$
	$D_{O_{2}}=2.652{\times 10}^{-5}{\left(T/333.15\right)}^{1.5}\times (101325/P)$	
	$D_{H_{2}O}=2.982{\times 10}^{-5}{\left(T/333.15\right)}^{1.5}\times (101325/P)$	

**Table 4:** Parameters for the electrochemical equations.

Electrochemical Properties	Value	Units
Reference molar concentration	$C_{H_{2}∕O_{2}}^{\mathrm{ref}}=40.88/40.88$	${\mathrm{mol}\;m}^{-3}$
Anode and cathode transfer coefficient	1	
Reference exchange current density	$i_{0,a/c}^{\mathrm{ref}}{=1\times 10}^{4}/0.05$	${A\;m}^{-2}$
ratio of reaction surface to CL volume	${a=1\times 10}^{5}$	${1\;m}^{-1}$

## Data Availability

Not applicable.
